# Sodium-Glucose Co-transporter-2 Inhibitors and Nephroprotection in Diabetic Patients: More Than a Challenge

**DOI:** 10.3389/fmed.2021.654557

**Published:** 2021-06-04

**Authors:** Michele Provenzano, Maria Chiara Pelle, Isabella Zaffina, Bruno Tassone, Roberta Pujia, Marco Ricchio, Raffaele Serra, Angela Sciacqua, Ashour Michael, Michele Andreucci, Franco Arturi

**Affiliations:** ^1^Chair of Nephrology, Department of Health Sciences, University “Magna Graecia” of Catanzaro, Catanzaro, Italy; ^2^Department of Medical and Surgical Sciences, University “Magna Graecia” of Catanzaro, Catanzaro, Italy; ^3^Unit of Internal Medicine, Department of Medical and Surgical Sciences, University “Magna Graecia” of Catanzaro, Catanzaro, Italy; ^4^Interuniversity Center of Phlebolymphology (CIFL), International Research and Educational Program in Clinical and Experimental Biotechnology at the Department of Surgical and Medical Sciences University Magna Graecia of Catanzaro, Catanzaro, Italy; ^5^Unit of Geriatric, Department of Medical and Surgical Sciences, University “Magna Graecia” of Catanzaro, Catanzaro, Italy

**Keywords:** CKD, type 2 diabetes, renal risk, cardiovascular risk, clinical trials, review, SGLT2i

## Abstract

Diabetic nephropathy is the most common cause of end-stage renal disease worldwide. Control of blood glucose and blood pressure (BP) reduces the risk of developing this complication, but once diabetic nephropathy is established, it is then only possible to slow its progression. Sodium-glucose cotransporter-2 inhibitors (SGLT2is) are a novel class of oral hypoglycemic agents that increase urinary glucose excretion by suppressing glucose reabsorption at the renal proximal tubule. SGLT2is lower glycated hemoglobin (HbA1c) without increasing the risk of hypoglycemia, induce weight loss and improve various metabolic parameters including BP, lipid profile, albuminuria and uric acid. Several clinical trials have shown that SGLT2is (empagliflozin, dapagliflozin canagliflozin, and ertugliflozin) improve cardiovascular and renal outcomes and mortality in patients with type 2 diabetes. Effects of SGLT2is on the kidney can be explained by multiple pathways. SGLT2is may improve renal oxygenation and intra-renal inflammation thereby slowing the progression of kidney function decline. Additionally, SGLT2is are associated with a reduction in glomerular hyperfiltration, an effect which is mediated by the increase in natriuresis, the re-activation of tubule-glomerular feedback and independent of glycemic control. In this review, we will focus on renal results of major cardiovascular and renal outcome trials and we will describe direct and indirect mechanisms through which SGLT2is confer renal protection.

## Introduction

Diabetes mellitus (DM) is a rapidly increasing disease. In 2030, it is estimated that about 7.7% of the total adult population will be affected by diabetes (1). An important complication of DM is chronic kidney disease (CKD), which occurs in about a third of diabetic patients, and represents one of the main causes of mortality and End-Stage-Kidney-Disease (ESKD) (2). The 2012 Kidney Disease Improving Global Outcomes (KDIGO) guidelines define CKD as chronic kidney damage characterized by albuminuria (≥ 30 mg/die) and/or reduced glomerular filtrate (eGFR) <60 ml/min/1.73 m^2^, persistent for at least 3 months (3). It has been widely demonstrated that eGFR <60 ml/min/1.73 m^2^ and the presence of albuminuria are two independent risk factors for all-cause and cardiovascular (CV) mortality (4, 5). The predictive ability of albuminuria and eGFR on cardiovascular events is similar to that of traditional risk factors, such as blood pressure (BP) or smoking habit. This lead some authors to consider CKD as one of the criteria defining a higher risk of future coronary events (6). Monitoring albuminuria is, thus, a crucial point of management of CKD patients, particularly in those also suffering from diabetes. In fact, Minutolo et al. (7) highlighted that the presence of mild or moderate albuminuria was associated with a higher risk of mortality and CV events in diabetic CKD compared with non-diabetic CKD patients, whereas the ESKD risk was correlated with the severity of albuminuria, regardless of the presence of diabetes. Diabetic nephropathy is an important microvascular complication that occurs in about 30% of patients with type 1 diabetes and 40% of those with type 2 diabetes (8). Diabetic nephropathy is related to the duration of diabetes and the quality of glycemic control. It is known that hyperglycemia plays a key role in developing kidney damage. Hyperglycemia causes glomerular hypertension and induces intra- and extra-cellular alterations, which lead to loss of glomerular barrier selectivity and expansion of the mesangial and interstitial matrix, resulting in glomerulosclerosis and interstitial tubular fibrosis (9). Over the past few decades, a number of randomized clinical trials have been carried out with the ambitious goal of reducing CV and renal risk in patients with CKD and type 2 diabetes (10). These studies have allowed to uncover the efficacy of drugs interfering with the Renin-Angiotensin-Aldosterone system, namely the Renin-Angiotensin-Aldosterone system inhibitors (RAASi), in relenting CKD progression and conferring CV protection to diabetic CKD patients (11, 12). Nevertheless, despite the wide and beneficial use of RAASi among clinicians of different specialties, these drugs have been shown to reduce but not delete the high risk for these patients (13–15). Hence, clinical research has focused resources and efforts in finding novel effective therapeutic strategies which may improve the standard-of-care. To this end, striking results have been provided by the sodium glucose co-transporter 2 inhibitors (SGLT2is), a new class of orally active drugs used in the management of type 2 diabetes that promotes glucose excretion in the kidney. SGLT2is not only improve fasting plasma glucose and HbA1c, but are able to induce BP reduction and weight loss and an improvement in kidney damage as well (16). The mechanisms by which SGLT2is promote such an improvement are not fully understood yet. This review provides an overview regarding the effects of these medications on the kidney and the most relevant findings derived from recent randomized clinical trials.

## SGLT2 Inhibitor

**Figure 1:**
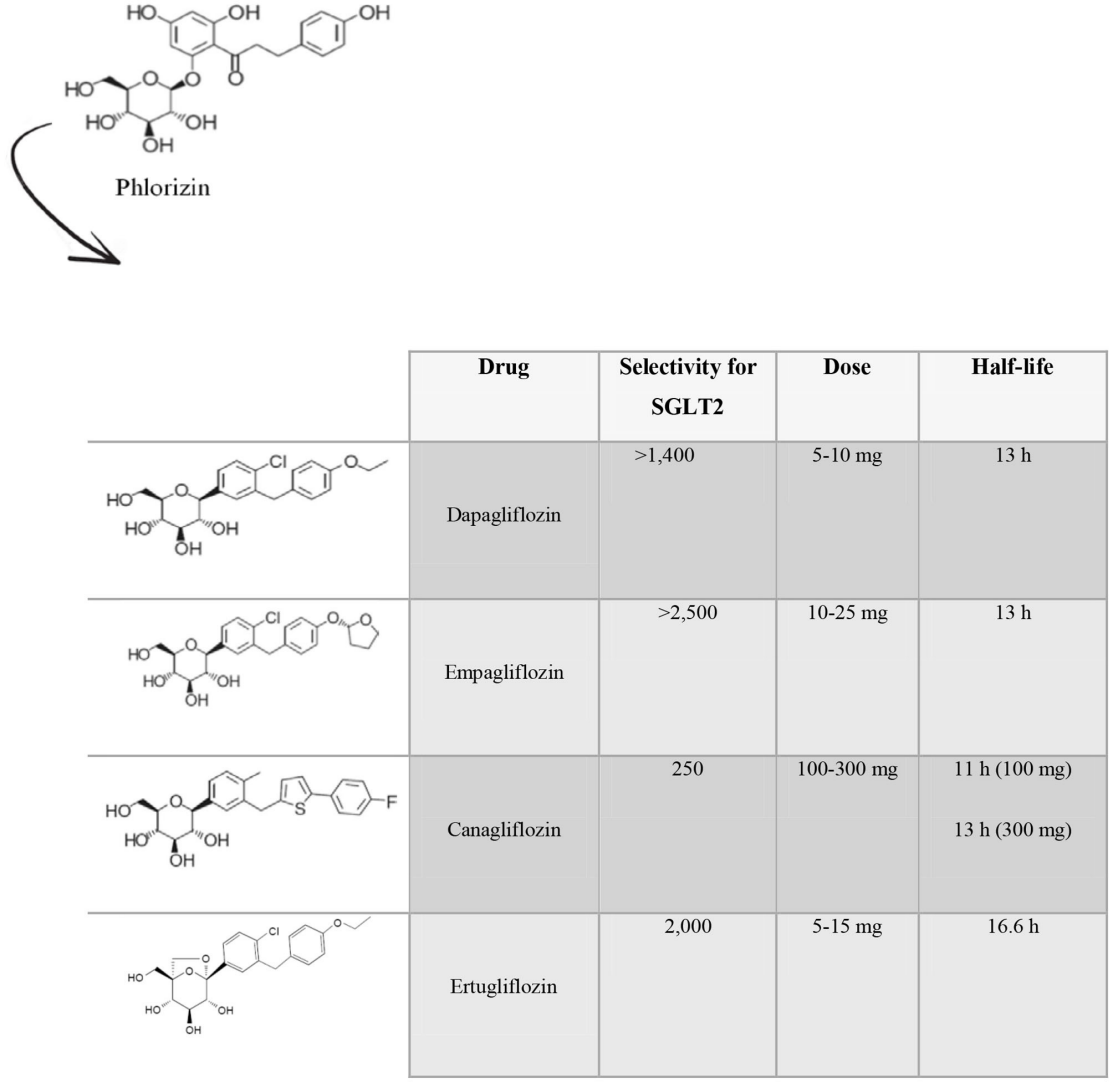
Molecular and pharmacokinetics characteristics of the principal SGLT2 inhibitors. Phlorizin was the first SGLT2 inhibitor discovered. Later, several gliflozins have been developed, keeping the active center of phlorizin and implementing structural changes in order to achieve a better bioavailability, selectivity for SGLT2 and decrease their side effects. Only four molecules have received Food and Drug Administration approval for the treatment of type 2 diabetes namely dapagliflozin, empagliflozin, canagliflozin, and ertugliflozin.

The first SGLT2i discovered was phlorizin, a molecule found in the root bark, leaves, shoots and fruit of the apple tree (17–19), whose use failed due to poor oral bioavailability, absence of selectivity for SGLT2 and gastrointestinal side effects (20). Several drugs called gliflozins were subsequently developed, retaining the active center of phlorizin and implementing structural changes in order to increase its bioavailability, its selectivity for SGLT2 and to decrease its side effects (21). To date, of the 9 existing SGLT2i molecules, only 4 have been approved by the Food and Drug Administration (FDA) for the treatment of type 2 diabetes namely dapagliflozin, empagliflozin, canagliflozin and ertugliflozin (Figure 1). The other ones are ipragliflozin, luseogliflozin, tofugliflozin, sotagliflozin, and remogliflozin. Dapagliflozin, the first SGLT2i approved for the treatment of type 2 diabetes (in Europe in 2012) (22), is a selective orally active SGLT2i (23), with a selectivity for SGLT2 > 1,400-fold greater than that for SGLT1 (24). The initial dose is 5 mg, which can be increased to 10 mg. Its half-life (T1/2) is 13 h (25, 26). Empagliflozin, approved in 2014, is the drug with the highest selectivity for SGLT2 over SGLT1 (>2,500-fold). The suggested dose is 10 mg and can be titrated up to 25 mg once daily (25). Its T1/2 is 13 h (24). Canagliflozin has 250-fold selectivity for SGLT2 over SGLT1. It is administered at an initial dose of 100 mg, that can be up titrated to 300 mg daily, with a T1/2 of 11 h and 13 h (for the 100 mg dose and the 300 mg dose), respectively (26). Ertugliflozin is the newest SGLT2i approved by the FDA. It has a 2,000-fold increase in selectivity for human SGLT2 over SGLT1 (27). The initial suggested dose is 5 mg once daily, and it can be titrated up to 15 mg once daily (25). Its elimination T1/2 is 16.6 h (28).

## Kidney Metabolism of Glucose: Physiological Role of the Sodium-Glucose Cotransporters

**Figure 2:**
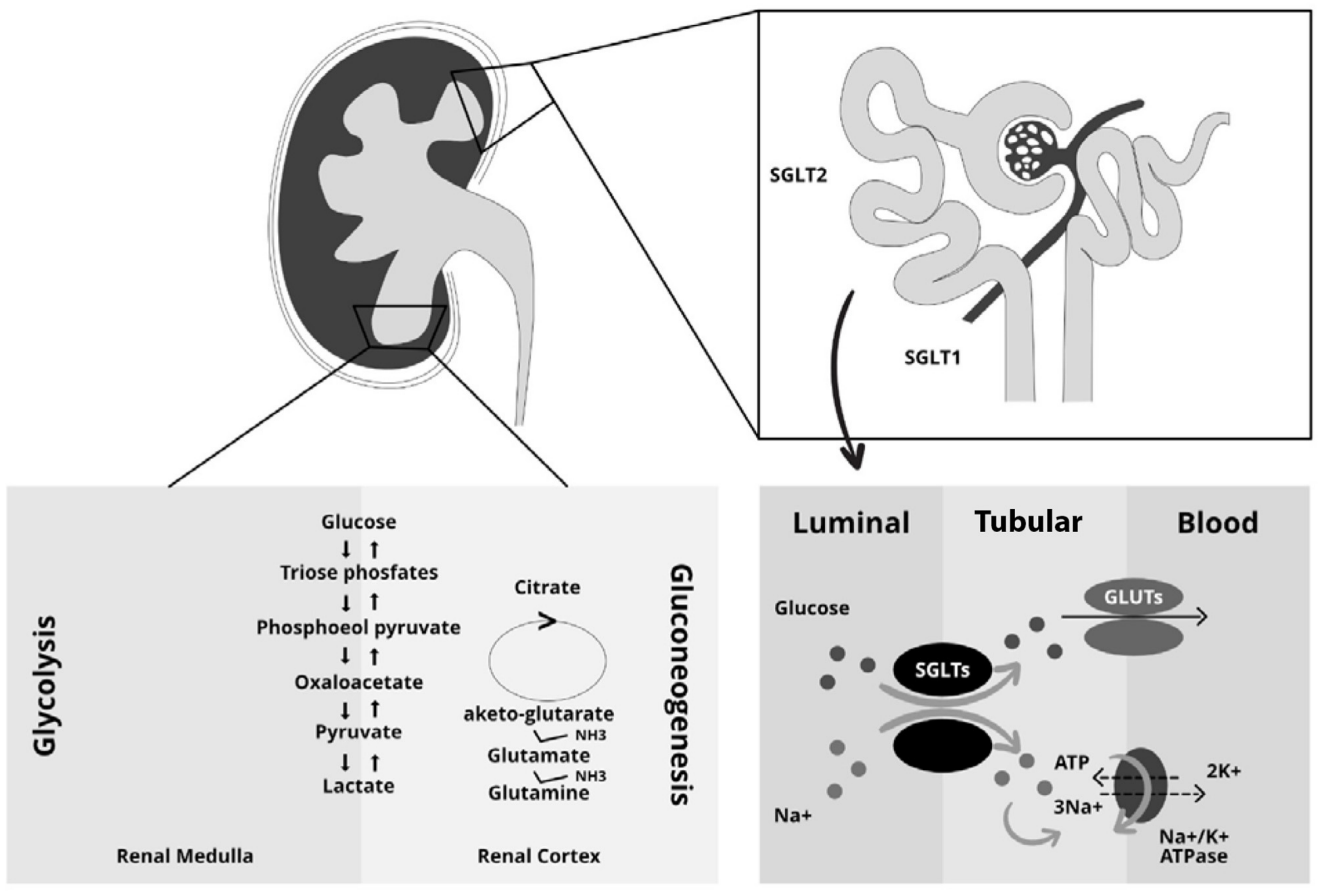
Central role of the kidney in glucose metabolism. The kidneys intervene in glucose homeostasis regulation through three main mechanisms: the glucose reabsorption via sodium co-transporters (SGLTs), the gluconeogenesis with endogenous glucose production and the utilization of glucose. The right side of the figure shows the mechanism of sodium and glucose reabsorption through the SGLT and GLUT transporters in the kidney. SGLTs, which are mainly located on the brush border (luminal side) of epithelial tubular cells, enables the transfer of both sodium and glucose from the lumen into the tubular cells. Sodium is transported along with glucose through the SGLTs. Next, glucose enters blood circulation through an active transport mechanism mediated by GLUTs, which are located on the basolateral membrane of the epithelial tubular cells. Sodium is actively exchanged between tubular cells and blood circulation through a Na+/K+/ATPase pump. The different phases of renal gluconeogenesis and glycolysis are depicted on the left side of the figure.

The kidneys play a key role in glucose homeostasis being involved in both glucose reabsorption *via* sodium co-transporters (SGLTs) and endogenous glucose production *via* gluconeogenesis as well as in glucose utilization (29–32). Reabsorption is mediated by SGLTs and Glucose transporters (GLUTs) (33); in particular SGLTs are expressed at the luminal brush border and GLUTs at the basolateral membrane of the epithelial cells (34). There are two types of SGLT co-transporters. The first one, SGLT2, is a high-capacity, low-affinity co-transporter; while the second one, SGLT1, is a high-affinity transporter expressed in the more distal part of the proximal tubules (35, 36). SGLT2 proteins are part of the SGLT family that include six different isoforms with different substrate specificity and localization. SGLT2 is mainly expressed in the kidney. In contrast, SGLT1 is mainly expressed in the intestine with a lower density being detectable in the kidneys, heart and skeletal muscles (37). In healthy individuals, all filtered plasma glucose is reabsorbed in the renal tubules, and the tubular maximum glucose absorptive capacity (TmG) is about 375 mg/min (29, 38, 39). When the filtered glucose exceeds the renal threshold, defined as glucose concentration at which the TmG is exceeded, glycosuria appears (40). In diabetic patients the TmG is higher than healthy individuals, this contributing to the worsening of hyperglycemia (41). The increase in absorptive capacity is due to upregulation or hyperactivation of GLUTs and SGLTs (30, 42). This mechanism, which may seem protective, leads to an increase in glycemia as it reduces the excretion of excess filtered glucose in the urine (30). The kidneys filter 160–180 g of glucose per day in healthy individuals (43) and ~97% of the filtered glucose is reabsorbed in the early proximal tubule by SGLT2; the remaining glucose is reabsorbed by SGLT1 in the late proximal renal tubule (44). The exact mechanism combines the reabsorption of sodium and glucose through the SGLTs (34–36). In particular, on the apical membrane of the renal tubule, sodium is transported along with glucose into the cell through the SGLTs and then the glucose enters in circulation through an active transport mechanism mediated by GLUT2, while sodium is actively exchanged through a Na+/K+ pump (31, 45). The sodium: glucose coupling ratio is 1:1 for SGLT2 and 2:1 for SGLT1 (46). The high affinity of SGLT1 for glucose together with the transport molar ratio of 2:1 leads to a complete reabsorption of the filtered glucose in the S3 segment of the proximal renal tubule. Another important mechanism contributing to renal glucose control is gluconeogenesis. In particular, after prolonged fasting, the kidney is able to produce about 20–25% of the glucose released in circulation, while, post-prandial renal gluconeogenesis increases by 100% and accounts for 60% of endogenous glucose release (47–49). This process could be explained by the use of endogenous glucose as a source for the constitution of liver glycogen (31). Renal gluconeogenesis, as well as hepatic gluconeogenesis, contributes to the worsening of hyperglycemia in diabetic patients (Figure 2) (31). Finally, the glucose utilization by kidneys is, in the fasting state, about 10% while in the post-prandial state increases by 3-fold. This mechanism is upregulated in diabetic patients in which there is increased renal glucose utilization (49–51). These drugs, causing glycosuria, lead to caloric loss and diuresis with a reduction of body weight and BP, and improve beta cell function, insulin sensitivity and consequently favor better glycemic control without hypoglycemia (50).

## Diabetic Kidney Disease: Role of SGLT2 Inhibitors

Initially, diabetic nephropathy is characterized by an increase of the GFR, a phenomenon known as glomerular or renal hyperfiltration (according to guidelines it is defined as a GFR of more than two standard deviations above the mean GFR of healthy subjects). Two major hypotheses are proposed to explain this phenomenon: the hemodynamic hypothesis, including the myogenic, neurogenic and hormonal factors which determine vasoconstriction of the afferent arteriole; and the tubular hypothesis, namely the early changes in diabetic kidneys involving proximal tubular reabsorption (52). Physiologically, tubules and glomeruli work together to maintain an adequate extracellular volume, through two mechanisms: glomerulo-tubular balance, that modulates renal proximal tubular reabsorption related to GFR changes, and tubule-glomerular feedback (TGF), which through the *macula densa* keeps constant the filtered load of solutes that reaches the renal distal tubule (53). Some studies in the rat showed that in the early stages of diabetes, TGF has a major role, in fact hyperglycemia causes proximal tubular hypertrophy, inducing an increase in renal proximal tubular reabsorption and in sodium/glucose cotransport. These alterations decrease the amount of solutes reaching the *macula densa* and so the TGF is deactivated, causing an increase in GFR (54). Data from the RIACE cohort shows that renal hyperfiltration is an independent predictor of death from any cause in patients with type 2 diabetes (55). In the study of Rigalleu et al. (56), kidney hypertrophy was correlated with disease progression in patients with overt diabetic nephropathy. Furthermore, renal tubular glucose active transport plays a key role in diabetic nephropathy (32). As previously described, glucose is mainly reabsorbed via the SGLT2 (57). SGLT2is reduce glucose reabsorption in the renal proximal tubules, thus promoting glucosuria. The degree of induced glucosuria is proportioned to the glycemic control, being greater in patients with higher circulating levels of glucose, as well as to the dose of the drug administered (58). The reduction in blood glucose levels is an important pharmacodynamic effect of SGLT2is. Moreover, this pattern of action is independent of insulin secretion and this means that these drugs also work in patients with reduced pancreatic β-cell function. Intriguingly, by blocking the SGLT2, these agents cause a decrease in sodium reabsorption. This pattern of action leads to two important consequences. The first one is the natriuretic effect, which results in reduction of intravascular volume and BP (59). The second one is the increase of sodium delivery to the *macula densa* that is followed by a re-activation of TGF, and a prompt reduction in single-nephron GFR. This effect is mediated by the vasoconstrictive adenosine and mitigates the renal hyperfiltration that is in turn responsible for deleterious long-term effects on the renal parenchyma (59).

SGLT2is have been shown to elicit several other nephroprotective effects. They improve endothelial dysfunction and reduced oxidative stress and inflammation, all of which are largely related to the effect of glucose on renal vascular and tubular cells (60). Furthermore, they have been shown to decrease the renal resistive index (RI), a marker of intrarenal vascular resistance that is also associated with a worse individual risk profile and prognosis. The effect of the SGLT2i dapagliflozin occurred after a short period (8-weeks) of treatment in a study enrolling type 2 diabetic patients (61, 62). Interestingly, SGLT2is seem to improve the regulation of extracellular matrix deposition, by decreasing the epithelial-to-mesenchymal transition, a mechanism which involves metalloproteinases and predisposes to renal fibrosis over time (63, 64). SGLT2is significantly lower albuminuria, thus reducing its toxic effects on renal tubules (65). The reduction in albuminuria levels exerted by the SGLT2is is largely dependent on the reduction of intraglomerular pressure (66). In fact, SGLT2is have positive effects on both the afferent and efferent arteriolar tone. Previous studies in patients with type 1 diabetes showed that empagliflozin lowers glomerular hyperfiltration through the increase of afferent renal resistance (67, 68) with a mechanism that may involve the release of adenosine and its effect on adenosine A1 receptors. In type 2 diabetes, the main mechanism by which SGLT2is reduce the hyperfiltration is more likely due to the post-glomerular vasodilation via the activation of TGF and the effect of adenosine on A2 receptors (66). Other beneficial mechanisms that lead to the reduction of albuminuria have been reported. In animal models, SGLT2is reduce blood levels and tissue expression of inflammation, oxidative stress and fibrosis (69, 70). Such non-hemodynamic effects may potentiate the hemodynamic effects on renal inflammation and fibrosis which is *per se* determined by the reduction in glomerular hyperfiltration. Altogether, although it is often complex to distinguish between the weight of hemodynamic and non-hemodynamic patterns, all these mechanisms confer a renoprotection and significantly reduce albuminuria.

## Renal Oxygenation

As previously discussed, glomerular hyperfiltration is a key pathogenetic step in the determination of diabetic kidney disease. It has been shown that renal hyperfiltration is associated with increased renal O_2_ consumption and with reduced availability of O_2_ in the renal cortex and medulla (71). This phenomenon has been attributed to the increase in reabsorption of Na^+^ via the SGLT that leads to a raised activity of the Na^+^/K^+^ATPase pump in the proximal kidney tubules. O'neill et al. have interestingly shown, in a rat model of diabetic kidney disease, that the inhibition of SGLT with the non-selective agent phlorizin reversed renal cortical hypoxemia via the reduction of glomerular hyperfiltration and sodium reabsorption (72). More specifically, phlorizin reduced glomerular filtration by 4 mL/min on average in diabetic rats included in this study and O_2_ consumption was significantly reduced in diabetic rats but not in control rats. Conversely, the increase of fractional sodium excretion caused an overload of Na^+^ to the distal part of the nephron raised O_2_ consumption and worsened hypoxia in the renal medulla. All these findings suggested a strict connection between glomerular filtration, Na^+^ uptake and renal hypoxia. The peculiar mechanism of action of SGLT2is may predispose to hypoxic damage of a portion of the renal parenchyma, namely the medulla, that is *per se* more susceptible to this pathogenic insult. Indeed, while the renal cortex physiologically receives enough O_2_ to supply the metabolic needs, the renal medulla is supplied with only about 10% of the total blood renal flow (73). Such low partial pressure of O_2_ in the medulla reflects limited regional blood flow that enables the generation of high medullary osmolality, required for the urine concentrating capacity. The degree of tubular activity, and thus the O_2_ consumption, is regulated by several substances including adenosine, nitric oxide and prostaglandins which modulate the distal tubular transport through complex mechanisms that are impaired in the presence of CKD. Moreover, the presence of type 2 diabetes *per se* contributes to the low oxygenation amount of renal parenchyma (74). As a consequence of the hypoxic effect on the renal medulla, SGLT2is causes an increase in hematocrit (75–77). In a randomized study evaluating the diuretic effects of dapagliflozin, hydrochlorothiazide (HCT) vs. placebo, Lambers Heerspink et al. found an increase of hematocrit by 2.2% in patients treated with dapagliflozin compared with those treated with placebo after 3 months of treatment (75). Moreover, the increase in hematocrit in this trial was also accompanied by an increase in the mean reticulocytes and serum erythropoietin (EPO), thus suggesting a role of enhanced erythropoiesis. Erythropoietin is released from peritubular interstitial cells in response to reduced parenchymal O_2_. The EPO gene is upregulated by the hypoxia-inducible factors (HIFs) a heterodimer composed of α and β subunits. Hypoxia prevents the proteasomal degradation of the HIF-α subunit. Hence, it has been hypothesized that SGLT2is, by reducing renal oxygenation at the corticomedullary junction, lead to the stabilization and accumulation of HIF-α, permeating its binding to the beta subunit and the induction of HIF-mediated genes (73, 77). Hence, SGLT2is seem to exert an opposite effect toward the cortical and medullary portion of the kidney. In a recent clinical study enrolling patients with Acute Kidney Injury (AKI) while taking SGLT2is, Darawshi et al. found that, in this specific patients population, the biomarkers of distal renal tubular injury such as serum and urine Neutrophil Gelatinase-Associated Lipocalin (NGAL) levels were higher than the levels found in patients without AKI, whereas biomarkers of proximal renal tubular injury namely serum and urine kidney injury molecule-1 (KIM-1) levels were not significantly different between the two groups (78). Such important findings suggest that SGLT2is may mitigate hypoxic injury in cortical parenchyma and, at the same time, may trigger hypoxic injury in the renal medulla, this being particularly in high risk conditions such as the presence of diabetes or CKD (or both clinical entities).

## SGLT2 Inhibitors and Acute Kidney Injury

The novel drug family of SGLT2is has rapidly captured the attention of clinicians given their efficacy in slowing CKD progression and in reducing CV risk in patients with CKD and diabetes. This notwithstanding, since their introduction in clinical practice several studies raised concerns regarding their safety, particularly with regards to the risk of AKI (79, 80). The mechanisms underlying the onset of AKI during treatment with SGLT2is are represented by their actions on glomerular hemodynamics, the effect of volume depletion and the hypoxic effect (73). The initiation of treatment with SGLT2is is followed by a variable eGFR reduction of 2–6 mL/min/1.73m^2^ lasting for about 2 weeks (81). This initial dip is followed by a subsequent reduction in eGFR decline over time (82–84). Hence, a biphasic trajectory of eGFR following SGLT2is start has been reported. The initial eGFR decline mainly reflects the reduction of glomerular hyperfiltration which is a crucial pathogenetic mechanism of diabetic kidney disease. This means that patients who present the initial dip in eGFR would be those who will experience the greater protection from eGFR decline in the long follow-up. Moreover, it has been shown that the biphasic trajectory is shared by almost all SGLT2is and it has been reported for both higher and lower basal eGFR levels (85, 86). Due to these pieces of evidence, De Nicola et al. hypothesized this initial dip of eGFR to be a “check-mark sign” of SGLT2is activity (80). Data surrounding the true incidence of AKI after SGLT2is are controversial. In a meta-analysis of randomized trials evaluating the adverse renal outcomes (eGFR decline up to renal failure) in patients with type 2 diabetes, dapagliflozin but not empagliflozin was associated with a high risk for adverse renal events compared with placebo (87). Conversely, in the CANVAS trial, canagliflozin was not significantly associated with the risk of AKI (88). The reports submitted to the FDA for the period March 2013-October 2015, signaled 101 cases of AKI during treatment with canagliflozin or dapagliflozin and principally occurred in patients with CKD, volume depletion, old age, concomitant use of diuretics, concomitant treatment with RAASi or non-steroidal anti-inflammatory drugs (89). However, data regarding the true incidence of AKI are limited and further studies are needed to clarify whether this phenomenon is present and whether it is associated with all SGLT2is or to a specific one.

## Extra-Kidney Effects of SGLT2 Inhibitors

The effects of SGLT2is are not limited only to glycemic control and/or to improve CKD. They have also shown extra-kidney beneficial effects that can also contribute to the improvement of renal hemodynamics.

### Cardiac Effects

Cardioprotective effects of SGLT2is have been evaluated in several cardiovascular outcome trials (CVOTs) (88, 90–92). Three SGLT2is, empagliflozin, canagliflozin, and dapagliflozin have so far been evaluated (88, 90–92). In patients with type 2 diabetes and established atherosclerotic CV disease, SGLT2is showed a significant reduction of major cardiovascular events (MACE), the composite of non-fatal myocardial infarction (MI), stroke and CV death (93). Moreover, these antidiabetic agents reduced by 30–35% the rates of hospitalization for heart failure, both in patients with and without CV disease (88, 90–92). The cardioprotective mechanisms of SGLT2is have been largely studied but, to date, not yet proven. They cannot be explained entirely by the glycemic control and the improvement of traditional CV risk factors (BP, body weight, lipid profile, arterial stiffness) as they take a long time to give cardiovascular protection (94, 95). Indeed, beneficial effects were observed after a short time interval, indicating the presence of a hemodynamic, rather than anti-atherosclerotic effect (96). Similar results were observed in a clinical trial evaluating the effect of dapagliflozin in patients with HF (96).

### Blood Pressure

In the renal proximal tubule, the inhibition of SGLT leads to natriuresis, osmotic diuresis and a consequent extracellular volume contraction. This results in a decrease of BP, which is more marked for higher baseline systolic BP values (93). Other factors implicated in the decrease of BP may be weight loss, sympathetic nervous activity reduction and inhibition of myocardial sodium-hydrogen exchanger 3 (NHE3), which modulates sodium reuptake (97) and seems to concur to oxidative stress and HF (98). Conversely, improvement in glycemic control seems not to affect BP.

### Uric Acid

SGLT2is increase renal uric acid excretion, thereby reducing serum uric acid concentrations (99). SGLT2is raise glucose concentration in the renal tubules, the site where glucose competes with urates for the Glucose transporter 9b (GLUT9b). This leads to a reduction in urate reabsorption (99). This reduction became modest or absent in patients with CKD (100).

### Ketone Bodies

SGLT2is cause a reduction in blood glucose levels which, in turn, lead to a shift in cellular metabolism. The result of such change is the increased use of fatty acid oxidation and lipolysis as energy sources. Lipid oxidation generates acetyl-CoA that is converted in ketone bodies when glucose oxidation is reduced. Lower plasma glucose level stimulates glucagon secretion and suppression of insulin production leading to an increase in the glucagon: insulin ratio. As previously discussed, SGLT2is induce natriuresis and volume depletion. It has been demonstrated that SGLT2is increase renal tubular reabsorption of acetoacetate from the proximal tubule during volume depletion, thus contributing to the pathogenesis of hyperketonemia (101, 102). Moreover, SGLT2is, like phlorizin, can decrease renal clearance of ketone bodies. These metabolic changes overall promote ketogenesis (103). Oxidation of ketone bodies produces more amounts of ATP per molecule of oxygen than glucose or fatty acids oxidation does and may provide a more efficient energy source for the myocardium (103). Moreover, ketones generate a minor production of reactive oxygen species (ROS) and therefore they can contribute to prevent mitochondrial dysfunction. In fact, enhanced production of ketone bodies has been proposed as one mechanism driving protection from cardiovascular death and HF observed during SGLT2is therapy. On the other hand, the increase in concentrations of ketone bodies leads to the feared diabetic ketoacidosis (DKA) (104).

### Oxidative Stress and Inflammation

Oxidative stress is involved in the development of diabetic complications and plays a key role in the progression of atherosclerosis (105, 106). SGLT2is have recently showed to protect against oxidative stress by direct and indirect mechanisms, such as glucose and free radical generation lowering. Prior studies reported that SGLT2is prevent mitochondrial dysfunction by improving redox state (107), they modulate expression and/or activity of pro-oxidant enzymes (108) and decrease advanced glycation end products (AGEs) generation by reducing glycaemia (109). In addition, SGLT2is may decrease oxidative damage also by inhibiting inflammatory pathways. Indeed, treatment with SGLT2is was associated with a suppression of serum inflammatory markers, such as IL-6 and Tumor Necrosis Factor-α (TNF-α) (110), and with a reduction of MCP-1, p65, toll-like receptor 4 andosteopontin expression (111).

### Arterial Stiffness and Endothelial Dysfunction

In several studies the chronic or acute use of SGLT2is improved arterial stiffness and endothelial dysfunction (7, 60, 112), independent of changes in BP.

### Adipokin Levels

A reduction in serum leptin levels and an increase in serum adiponectin, which has an anti-inflammatory effect, has been observed during SGLT2i therapy (110). To date, it is unclear whether SGLT2is alters adipokine levels with direct or indirect action in adipose tissue.

### Clinical Trials on SGLT2is and Renal Outcomes

Several placebo-controlled clinical trials have demonstrated that SGLT2is could prevent the development of chronic kidney disease or decrease the progression of kidney disease in patients with type 2 diabetes. The nephroprotective effects of SGLT2is have been demonstrated in five major CVOTs: EMPA-REG OUTCOME (empagliflozin), CANVAS and CANVAS-R (canagliflozin), DECLARE-TIMI 58 (dapagliflozin) and VERTIS CV (ertugliflozin). All these trials were designed to investigate CV outcomes in patients with type 2 diabetes and established atherosclerotic CV disease or multiple CV risk, and the renal endpoints were evaluated only as secondary outcome. Most recently, two clinical trials, the CREDENCE trial (canagliflozin) and DAPA-CKD trial (dapagliflozin), were designed to investigate renal outcomes as the primary endpoint. In addition, several clinical studies evaluated the effects of SGLT2 on eGFR and urinary albumin to creatinine ratio (UACR) in patients with type 2 diabetes.

### Empagliflozin

EMPA-REG OUTCOME was a randomized, double-blind, placebo-controlled trial involving patients with type 2 diabetes, almost all with established CVD. Patients had a significant history of atherosclerotic heart disease including MI, coronary artery bypass grafting and/or multiple vessel disease. The mean eGFR at baseline was 74.1 mL/min/1.73 m^2^ and the median UACR was 18 mg/g.

The EMPA-REG OUTCOME trial showed that compared with placebo, empagliflozin was able to improve a primary composite endpoint including CV death, MI or stroke. Moreover, it improved all-cause mortality and significantly reduced the rates of hospitalization for HF. Composite renal outcomes in the EMPA-REG OUTCOME trial included incident or worsening nephropathy and incident albuminuria (86). Worsening nephropathy (defined as progression to macroalbuminuria, doubling of serum creatinine, initiation of renal replacement therapy or death from renal causes) was significantly lower in patients treated with empagliflozin when compared with placebo.

Furthermore, *post-hoc* analysis of the EMPA-REG-OUTCOME trial showed that empagliflozin significantly slowed the decline in eGFR (113). Interestingly, the results observed in this study were obtained also when only modest reduction of A1c in the treatment arm were observed. Moreover, it is not clear if renal benefits observed in this population with high cardiovascular risk may also be obtained in patients with low cardiovascular risk and/or without diabetes. At present there is an ongoing study assessing the renal effects of Empagliflozin in patients with CKD with or without type 2 diabetes, namely The Study of Heart and Kidney Protection With Empagliflozin (EMPA-KIDNEY, Clinical-Trials.org identifier NCT03594110).

### Canagliflozin

Similar results were observed with canagliflozin in the CANVAS and CANVAS RENAL (CANVAS Program) studies. These studies were designed to evaluate the efficacy and the safety of canagliflozin in a population of diabetic patients with different levels of CV risk. The primary composite endpoint included CV death, non-fatal MI or non-fatal stroke. The rate of the primary outcome was lower in patients in treatment with canagliflozin than with placebo (HR, 0.86; 95% CI, 0.75–0.97; *P* < 0.001 for non-inferiority; *P* = 0.02 for superiority). When death from any cause and death from cardiovascular causes, were evaluated individually, significant differences between two arms of treatment were not found. Canagliflozin significantly reduced the rates of hospitalization for HF [HR, 0.67 (95% CI, 0.52–0.87)] and the benefit appeared to be similar for patients with reduced ejection fraction (HFrEF) and patients with preserved ejection fraction (HFpEF). Renal outcomes in the CANVAS trials were progression of albuminuria or regression of albuminuria and the renal composite comprising a 40% reduction in eGFR sustained for at least two consecutive measures, the need for renal-replacement therapy (dialysis or transplantation), or death from renal causes. Progression of albuminuria was defined as more than a 30% increase in albuminuria and a change from either normal-albuminuria to microalbuminuria or macroalbuminuria or from microalbuminuria to macroalbuminuria. Regression of albuminuria was defined using criteria comparable to those defined for category progression. Progression of albuminuria occurred less frequently among patients in therapy with canagliflozin than among those assigned to the placebo arm and the effects were greater in CANVAS-R (HR, 0.64; 95% CI, 0.57–0.73) than in CANVAS (HR, 0.80; 95% CI, 0.72–0.90) (*P* = 0.02 for homogeneity). Similarly, regression of albuminuria occurred more frequently among those assigned to canagliflozin than among those assigned to placebo. The composite renal outcome was also reduced in patients treated with canagliflozin than among those in the placebo group. No significant difference in this outcome was seen between CANVAS and CANVAS-R (88).

The EMPA-REG OUTCOME, CANVAS, and CANVAS-R trials, as well as the DECLARE-TIMI-58 (Dapagliflozin Effect on Cardiovascular Events-Thrombolysis in Myocardial Infarction 58) were clinical trials in which efficacy and safety of SGLT2is inhibitors on CV outcomes were evaluated in patients with type 2 diabetes and established or at high risk of atherosclerotic heart disease. Most patients enrolled in these trials showed variable degrees of diabetic kidney disease but generally with an estimated glomerular filtration rate (eGFR) of >60 mL/min/1.73m^2^ whereas the safety and efficacy of SGLT2is in patients with more severe CKD still remain undefined.

The CREDENCE (Canagliflozin and Renal Events in Diabetes with Established Nephropathy Clinical Evaluation) trial was a double-blind, randomized trial, that enrolled patients with T2D and albuminuric chronic kidney disease to receive canagliflozin or placebo. All the patients had an eGFR of 30 to <90 ml per minute per 1.73 m^2^, and albuminuria (ratio of albumin [mg] to creatinine [g]) of >300–5,000 and were treated with renin–angiotensin system blockade. The CREDENCE trial represents therefore the first dedicated renal outcomes trial with an SGLT2 inhibitor. Indeed, the primary outcome was a composite of end-stage kidney disease (dialysis for at least 30 days, transplantation, or a sustained estimated GFR of <15 ml per minute per 1.73 m^2^, for at least 30 days), a doubling of the serum creatinine level, or death from renal or CV causes. The CREDENCE trial has been prematurely stopped after achievement of the renal primary endpoint composite during the interim analysis. The relative risk of the primary composite outcome was significantly lower in patients in treatment with canagliflozin than in the placebo group (HR, 0.70; 95% CI, 0.59–0.82; *P* = 0.00001) (92).

Similarly, the relative risk across renal components, including the doubling of the serum creatinine level and the outcome of dialysis, kidney transplantation, or renal death was significantly lower in the canagliflozin group than in the placebo group (HR, 0.60; 95% CI, 0.48–0.76; *P* < 0.001 and HR, 0.72; 95% CI, 0.54–0.97, respectively). The nephroprotective effects of canagliflozin were also observed in the subgroup of patients with more advanced kidney disease and very low eGFR (eGFR 30–45 mL/min/1.73 m^2^) (HR, 0.75; 95% CI, 0.59–0.95). Moreover, patients treated with canagliflozin also had a lower risk of several secondary CV outcomes including the composites of CV death or hospitalization for HF (HR, 0.69, 95% CI, 0.57–0.83; *P* < 0.001), CV death, MI, or stroke (HR, 0.80, 95% CI, 0.67–0.95; *P* < 0.01) and hospitalization for HF (HR, 0.61, 95% CI, 0.47–0.80; *P* < 0.001).

Interestingly, these results have been obtained although very low and non-significant differences in blood glucose levels were observed between the group treated with canagliflozin and the placebo. This supports the hypothesis that the effects induced by SGLT2is on renal function are likely independent of glucose levels. Moreover, the observed benefits were obtained in patients that received stable doses of angiotensin-converting enzyme inhibitor (ACE-i) or angiotensin II receptor blocker (ARB) therapy, which are other medications that can induce nephroprotective effects in type 2 diabetes (92).

### Dapagliflozin

Dapagliflozin has been the first SGLT2 inhibitor approved for the treatment of patients with type 2 diabetes. Safety and efficacy of dapagliflozin on major adverse cardiovascular events (MACE) were evaluated in the DECLARE-TIMI-58, a randomized, double-blind, placebo-controlled trial including people with type 2 diabetes and atherosclerotic CV disease or multiple CV risk factors. In this study, Dapagliflozin resulted in a significantly lower rate of CV death and hospitalization for HF than placebo but failed to achieve superiority to reduce MACE (HR 0.93, 95% CI 0.84–1.03; *P* < 0.001 for non-inferiority and *P* = 0.17 for superiority). Similarly, no significant difference in the rate of death from any cause was observed between the two groups. Dapagliflozin showed a significant decrease in the renal composite outcome (>40% eGFR reduction, new ESKD or renal or cardiovascular death) when compared with placebo (HR 0.76, 95% CI 0.67–0.873) (91).

More recently, the efficacy and safety of dapagliflozin on progression of chronic kidney disease has been evaluated in patients with chronic kidney disease and with and without type 2 diabetes. In the Dapagliflozin and Prevention of Adverse Outcomes in Chronic Kidney Disease (DAPA-CKD) study, patients with and without diabetes, an eGFR of 25 to 75 mL/min/1.73 m^2^ and UACR of 200–5,000 milligrams, were enrolled. All the participants received a stable dose of an ACE-i or ARB. The primary outcome was a composite of a sustained decline in the eGFR of at least 50%, the onset of end-stage kidney disease (dialysis for at least 28 days, transplantation, or a sustained eGFR of <15 ml per minute per 1.73 m^2^, for at least 28 days), or death from renal or CV causes. Patients who received dapagliflozin had a significantly lower risk of the primary composite outcome than those that received placebo (HR, 0.61; 95% CI, 0.51–0.72; *P* < 0.001) and the effects were similar in patients with type 2 diabetes (HR, 0.64; 95% CI, 0.52–0.79) and without type 2 diabetes (HR, 0.50; 95% CI, 0.35–0.72). Moreover, patients in dapagliflozin group also had a lower risk for the composite of CV death or hospitalization for HF (HR, 0.71, 95% CI, 0.55–0.92). As previously reported in other studies with SGLT2 inhibitors, also in the DAPA-CKD trial in the first 2 weeks of treatment, there was a greater reduction in eGFR in patients in treatment with dapagliflozin than in placebo group, but thereafter the decline in the eGFR was slower in the dapagliflozin arm than in the placebo arm (114). The benefits of dapagliflozin on some markers of renal function (eGFR and/or urinary UACR) have also been explored in other clinical studies. The outcomes and the results of the DERIVE, DELIGHT, and DIAMOND studies are summarized in the Table 1.

**Table 1:** Studies comparing risk renal outcomes among patients with CKD treated with SGLT2i.

**Study**	**Population**	**Sample size**	**Intervention**	**Outcome**	**Results**
EMPA-REG OUTCOME study (86)	T2DM at high risk for cardiovascular events	7,020 empagliflozin 10 mg (*n* = 2,345), 25 mg (*n* = 2,342), or matching placebo (*n* = 2,333)	empagliflozin 10 mg vs. empagliflozin 25 mg vs. placebo	Incident or worsening nephropathy (progression to macroalbuminuria, doubling of the serum creatinine level, initiation of renal-replacement therapy, or death from renal disease) and incident albuminuria.	Improvement of incident or worsening nephropathy (such as doubling of serum creatinine) for empagliflozin vs. placebo (12.7% vs. 18.8%, HR 0.61, 95% CI 0.53–0.70; *p* < 0.001), decrease of progression to macroalbuminuria (11.2 vs. 16.2%, *p* < 0.001)
CANVAS study (88)	T2DM at high risk for cardiovascular events	10,142 canagliflozin (*n* = 5,795) vs. placebo (*n* = 4,347	canagliflozin 100, 300 mg vs. Placebo	Progression of albuminuria	Canagliflozin was also associated with a lower rate of progression of albuminuria (*p* < 0.05)
DECLARE-TIMI 58 (91)	T2DM and established CV disease or risk factors for atherosclerotic CV disease	17,160 dapagliflozin 10 mg (*n* = 8,582) or placebo (*n* = 8,578).	dapagliflozin 10 mg vs. Placebo	≥40% decrease in eGFR to <60 mL/min/1.73 m^2^ or new end-stage renal disease or death from renal/CV cause	The incidence of the renal composite outcome was 4.3% in the dapagliflozin group and 5.6% in the placebo group (hazard ratio, 0.76; 95% CI, 0.67–0.87).
VERTIS CV study (115)	T2DM and established CV disease	8,246 ertugliflozin 5 mg (*n* = 2,752), 15 mg (*n* = 2,747), or placebo (*n* = 2,747)	Ertugliflozin 5, 15 mg vs. Placebo	Renal death or dialysis/transplant or doubling of serum creatinine from baseline	Renal composite (renal death, dialysis/transplant, doubling of serum creatinine) not achieve statistical significance (3.2 vs. 3.9%, *p* = 0.08.
CREDENCE study (92)	CKD and T2DM	4,401 canagliflozin 100 mg daily (*n* = 2,202) or placebo (*n* = 2,199)	canagliflozin Vs. Placebo	Composite of ESRD (dialysis, transplantation, or sustained estimated GFR of <15 mL/min/1.73 m^2^), doubling of the serum creatinine, or death from renal or cardiovascular causes.	ESRD, doubling of serum creatinine, renal or cardiovascular (CV) death, for canagliflozin vs. placebo, was 43.2 vs. 61.2 per 1,000 patient-years (P-Y) (*p* = 0.00001)
DELIGHT study (116)	T2DM and chronic kidney disease of moderate to severe grade	1,187 dapagliflozin group (*n* = 145), dapagliflozin–saxagliptin group (*n* = 155), placebo group (*n* = 148)	dapagliflozin 10 mg only, dapagliflozin 10 mg and saxagliptin 2.5 mg, or placebo once-daily	Worsening nephropathy and progression of albuminuria	Dapagliflozin with or without saxagliptin, in addition to ACE-i or ARBs, slows the progression of kidney disease in patients with diabetes and chronic insufficiency from moderate to severe.
CANTATA-SU (117)	T2DM patients already treated with metformin	1,450 Canagliflozin 100 mg (*n* = 483), Canagliflozin 300 mg (*n* = 485), Glimepiride (*n* = 482)	canagliflozin 100 mg or 300 mg/day vs. glimepiride 6–8 mg/day	Decrease in eGFR and progression of albuminuria.	It showed a reduction in eGFR: −0.5 (canagliflozin 100 mg), −0.9 (canagliflozin 300 mg), −3.3 (glimepiride) mL/min/1.73 m^2^ at 2 years; and a reduction of albuminuria: −31.7% (canagliflozin 100 mg), −49.3% (canagliflozin 300 mg) relative to glimepiride.
DERIVE study (118)	Type 2 diabetes and chronic kidney disease in stage IIIA	302 dapagliflozin 10 mg (*n* = 160) or placebo (*n* = 161)	dapagliflozin vs. placebo	This study assessed the efficacy and safety of dapagliflozin 10 mg vs. placebo in patients with T2DM and moderate renal impairment (estimated glomerular filtration rate [eGFR], 45–59 mL/min/1.73 m^2^; chronic kidney disease [CKD] stage 3A)	This study underlines the positive benefit and poor risk profile of dapagliflozin for the treatment of patients with T2D and CKD 3A.
DIAMOND study (119)	CKD in patients with non-diabetic kidney disease	58 dapagliflozin then placebo (*n* =27) and placebo then dapagliflozin (*n* =26)	dapagliflozin vs. Placebo	Change in 24-hr proteinuria and GFR in patients with non-diabetic kidney disease	This study showed that 6-week treatment with dapagliflozin did not affect proteinuria in this sample, but induced an acute and reversible reduction of eGFR.
DAPA-CKD study (85)	CKD in patients with non-diabetic kidney disease	4,304 dapagliflozin 10 mg (*n* = 2,152) placebo (*n* = 2,152)	Dapagliflozin vs. Placebo	Occurrence of one of the components of the composite: ≥50% sustained decline in eGFR or reaching End Stage Kidney Disease or CV death or renal death	The risk of a composite endpoint was significantly lower with dapagliflozin than with placebo, regardless of the presence or absence of T2DM.

### Ertugliflozin

Efficacy and safety of ertugliflozin on cardiovascular and renal outcomes were assessed in the eValuation of ERTugliflozin EffIcacy and Safety Cardiovascular Outcomes Trial (VERTIS CV), a multicenter, randomized, double-blind, placebo-controlled trial including people with T2D and established atherosclerotic cardiovascular disease. The primary composite outcome included CV death, non-fatal MI or non-fatal stroke. Secondary outcomes were a composite of death from CV causes or hospitalization for HF; death from CV cause and a composite of death from renal causes, doubling of the serum creatinine level or renal replacement therapy. Unlike previous CV outcomes trials with SGLT2 inhibitor, ertugliflozin was non-inferior to placebo with regard to the primary composite outcome but failed to achieve superiority to reduce major adverse CV events (death from cardiovascular causes, non-fatal MI or non-fatal stroke). Similarly, no significant benefit was observed in the secondary composite renal outcome (115, 120). These data are in contrast with those obtained in other trials with SGLT2is, although the population of patients with atherosclerotic cardiovascular disease enrolled in VERTIS-CV study was broadly similar to those of previous trials performed with other SGLT2is. However, recently, has been demonstrated that when used in addition to standard of care medications, ertugliflozin is associated with a decrease in the risk of a sustained 40% decline in eGFR, with preservation of eGFR over time and a reduction of UACR in individuals with established atherosclerotic CVD and type 2 diabetes (121).

The results of major CVOTs, renal outcomes trials and clinical studies are summarized in Table 1.

## Summary Results of the Clinical Trials Assessing the Efficacy of SGLT2is on Renal Endpoints

A number of large clinical trials have demonstrated improvement in renal outcome in high-risk patients with CKD and DM (88, 90–92, 114). The EMPA-REG, CANVAS, and DECLARE studies have shown as positive class-effects of the SGLT2is a significant reduction in albuminuria progression and preservation of eGFR decline over time (88, 90–92). The CREDENCE trial was specifically designed to evaluate renal outcomes in diabetic kidney disease patients. This study showed that, when added to the standard nephroprotective treatment, namely the maximum tolerated RAAS inhibition, canagliflozin exerted a striking 30% risk reduction for the renal endpoint (composite of ESKD, that is, dialysis for at least 30 days, transplantation, or a sustained eGFR of <15 mL/min/1.73 m^2^ for 30 days, doubling of the serum creatinine for at least 30 days, or death from renal or cardiovascular disease). More importantly, this effect was independent of the baseline eGFR, being true also for patients with more advanced CKD (92). Once again, also in the CREDENCE trial, the long-term risk reduction was associated with a significant reduction in albuminuria in the canagliflozin group. The DAPA-CKD study extended these evidences to the more general population of CKD, with or without diabetes, testifying that SGLT2is act on pathophysiologic patterns that are active in CKD *per se* (114).

## Future Perspectives: The Use of SGLT2is in the Personalized Medicine Era

The therapeutic *armamentarium* for the management of patients with CKD is undergoing an important revolution. If the early 2000's was the period of the demonstration of efficacy of RAASi agents in lowering CV and renal risk in CKD patients, then the 2020s' have been marked to be another step forward (10). During this period, several randomized studies have been completed and published (114, 122, 123). The Study of Diabetic Nephropathy with Atrasentan (SONAR) showed that the endothelin receptor antagonist (ERA) atrasentan confers protection against renal events (ESKD/doubling of serum creatinine) in patients with CKD and type 2 diabetes. The risk for the renal endpoint in the SONAR trial was 35% lower in the atrasentan arm compared with placebo (122). Furthermore, the finerenone in Reducing Kidney Failure and Disease Progression in Diabetic Kidney Disease (FIDELIO-DKD) study evaluated the efficacy of finerenone, a novel non-steroidal Mineralocorticoid Receptor Antagonist (MRA), vs. the onset of both renal and CV events (123). Finerenone, in the FIDELIO study, caused a significant CV and renal risk reduction and also showed the great advantage of being associated with a low rate of adverse events. None of the patients included in the finerenone arm had to discontinue the treatment due to hyperkalemia, which is a feared, potential life-threatening adverse event mainly attributed to the classic MRAs (e.g., spironolactone, eplerenone) (124). Hence, a great effort has been made to expand the therapeutic opportunities for reducing the high burden and residual risk of CKD patients (125–127). Interestingly, both the MRA and ERA agents show synergistic and additive effects if combined with the SGLT2is. With respect to ERA, they suffer a major adverse event: namely fluid (water and sodium) retention due to the stimulation of ENaC channels in the renal collecting duct. This effect is opposed by the SGLT2is that increase natriuresis in renal tubules. For this reason, a new clinical trial has been started combining the ERA zibotentan and the SGLT2i dapagliflozin in CKD patients (ZENITH trial: Protocol D4325C00001). ERAs and SGLT2is may potentiate their nephroprotective effects, such as the albuminuria reduction, mitigating the adverse events of each other. Moreover, MRA, ERA, RAAS is and SGLT2is share similar beneficial effects on renal parenchyma, reducing the oxidative stress, inflammation and fibrosis over time. Thus, one interesting future perspective would be to start more studies testing whether the combination of more drugs (including SGLT2is) with different mechanisms of action would warrant a better prognosis in CKD patients and what type of CKD patients would benefit from these combinations. Such a strategy may overcome the limit of current nephroprotective interventions and the high residual CV and renal risk of CKD patients (128). Moreover, although SGLT2is determine an initial dip of eGFR (check-mark sign) and a significant reduction in albuminuria, the magnitude of the short-term eGFR decline and the thresholds of albuminuria reduction that are associated with a protection in the long term are still undetermined. This initial eGFR dip is not only manifest after SGLT2is initiation but also as a hemodynamic response to low salt diet, RAAS inhibition, and antihypertensive treatments. Nevertheless, according to the first analysis the relationship between the eGFR dip and the following normalization of eGFR slope seems to be stronger for SGLT2is than for the other pharmacological and non-pharmacological treatments (80). Future studies should provide more insights into this topic given that diabetic kidney disease patients are likely to be at lower risk of hard renal endpoints mainly after a positive response in terms of albuminuria and eGFR during the 1st weeks of treatment, as shown in previous positive and even negative randomized trials from the past decades (129). SGLT2is would also be part of the novel trial designs or personalized medicine. The future direction will consider, with growing interest, to evaluate the drug's efficacy in small trials of similar patients (e.g., CKD patients with IgA Nephropathy or Membranous Nephropathy), rather than large trials including all patients with general eGFR/albuminuria thresholds. Examples of the new clinical trials designs are represented by the Master Trials Protocol (MTP) (130). These include the “Umbrella” trials, where patients are assigned to different treatments on the basis of the presence of a specific biomarker or other individual features. Contrariwise, MTP could be planned to evaluate a single treatment in different diseases, e.g., by including CKD patients with glomerular disease, DKD, hypertensive nephropathy as primary renal disease. These latter designs are defined as “Basket” trials. A third type, even more sophisticated than MTP, are the “Platform” trials (131). The Platform trial has a standing structure, with no predefined stopping date (hence the diction “Eternal” trial), during which patients can be started or withdrawn with a specific treatment without leaving the protocol in case the first treatment used is not effective. At the trial start, patients are included in an experimental cohort, namely a large database, whether they meet general inclusion criteria following the principle that disparate trials, with different treatments and outcomes, will be started. After the inclusion, patients are screened on the basis of clinical features, biomarkers or any other potential relevant information, including histology. Next, patients with strictly similar characteristics are randomized to an experimental treatment vs. the standard-of-care. If the treatment is not able to provide any benefit it is stopped and patients would be assigned to another treatment in the future. Conversely, the new treatment could replace the standard-of-care if it has been shown to improve the outcomes in that subgroup of patients. The advantages of MTP, in reference to traditional RCT, are that these studies enable to evaluate what is the best treatment for a disease by comparing multiple treatments as well as to find the best drug combination for a specific subgroup of patients with possibly the same mechanisms of the disease. Hence, the current general tendency is to include in randomized studies a small number of patients with similar characteristics rather than a large number of patients with more general inclusion criteria. These novel designs have been prompted in nephrology, and a “CKD-Platform,” the Global Kidney Patient Trials Network (ClinicalTrials.gov Identifier: NCT04389827) has been started in 2020 including 140 centers worldwide to identify patients eligible for clinical trials and to optimize treatment of CKD patients. SGLT2is, given their excellent results provided thus far, will be the best candidate to enter these novel trial strategies in the future.

## Conclusion

The SGLT2is are a new class of orally active drugs used in the management of type 2 diabetes that promotes glucose excretion in the kidney. SGLT2is not only improve fasting plasma glucose and HbA1c but are able to induce blood pressure reduction and weight loss, improvement of CV outcomes and an improvement in chronic kidney disease. The nephroprotective effects of SGLT2is have been demonstrated in several major intervention studies. However, all these trials were designed to investigate CV outcomes in patients with type 2 diabetes and established atherosclerotic CV disease or multiple CV risk and the renal endpoints were evaluated only as a secondary outcome. Most recently, two clinical trials, the CREDENCE trial (canagliflozin) and DAPA-CKD trial (dapagliflozin), were designed to investigate renal outcomes as primary endpoint.

These studies have demonstrated that SGLT2is are able to delay renal disease progression in patients with and without type 2 diabetes and chronic kidney disease.

Interestingly, the effects induced by SGLT2is on renal function are likely independent of glucose levels and thus these medications could also be used in patients with CKD without type 2 diabetes. However, beyond the important results reported thus far, future studies should be performed to clarify the mechanisms of cardiorenal protection of SGLT2is. Clinical studies assessing how to combine SGLT2is with other nephroprotective treatments are also eagerly expected.

## Author Contributions

MP, MCP, MA, and FA: conceptualization, validation, and formal analysis. MP, MCP, and FA: methodology, writing—review and editing, and supervision. MP, MCP, IZ, BT, RP, MR, RS, AS, AM, MA, and FA: investigation, data curation, and writing—original draft preparation. All authors contributed to the article and approved the submitted version.

## Conflict of Interest

The authors declare that the research was conducted in the absence of any commercial or financial relationships that could be construed as a potential conflict of interest.
